# Some Unpleasant Truths about Food and Luxuries

**Published:** 1889-11-30

**Authors:** 


### Some Unpleasant Truths About Food and Luxuries.

*(From an Occasional Correspondent.)*

Although legislation has of late years done much to prevent the adulteration of what we eat and drink, yet there are to-day sad tricks played upon us in connection with the food that supports us, the drinks that cheer us, and the drugs that are intended to put us to rights when we get out of sorts. As a matter of fact, almost every article that can be named that will pay to adulterate, undergoes this peculiarly British process. There are but few exceptions to the rule. Adulterations have regard to quality, and to colour, and to smell, but in every case the motive is gain. The greater the pungency of some articles, the higher the colour of others, the greater their marketable value. No matter that they are thereby deteriorated, no matter though these foreign substances are highly deleterious. The question is not, Will these articles, when they are dressed, be conducive to the health of the consumer? but, Will they enable the producer and manufacturer to grow more speedily rich?

To begin with tea. It is adulterated largely with exhausted tea-leaves, leaves other than those of tea, British and foreign. Among the former, those of sycamore, horse-chestnut, and plum; with lye-tea, paddy husk, sand, and starch; with astringent matters, such as kino and catechu; and coloured with plumbago or blacklead, gum indigo, Prussian blue, turmeric, Chinese yellow, China clay, French chalk, and flavoured with sulphate of iron, and other things. Coffee is no better off, as chicory is very largely used in it. Then we find roasted wheat, rye, and potato flours, roasted beans, mangel wurzel, acorns, burnt sugar or black jack, venetian red, red clay, damaged dog biscuits, peas coloured with reddle, bark from tanyards, logwood dust, mahogany dust. The adulterations are generally to be found in the ground coffee, but the berries are sometimes mixed with chicory compressed into shape resembling coffee berries, and in 1850 a Liverpool firm actually took out a patent for this purpose. No sensible housekeeper will purchase ground coffee for domestic use. Lump sugar is purer than the raw sugar. In brown sugar there are found fragments of cane nearly always, these being frequently so minute as to be visible only with the microscope—disgusting insects (the sugar mites) acari, sporules, and filaments of fungi and grape sugar are also present, often in great quantity. Then there is sand, plaster of Paris, chalk, glucose, and—as an accidental impurity—lead. Fortunately, the ordinary loaf sugar is rarely adulterated, and it contains no "mites," owing to the process of refinement to which it is subjected, and the fragments of cane are also separated by the filtration through charcoal, a part of the refining process.

The adulteration of milk is too well known to say much about it; but, in addition to the "wooden cow," starch, dextrine or gum, annatto, chalk, and emulsion of seeds, are all met with, though rarely. Frauds, such as skimming the milk, or first skimming and then watering, are not strictly adulterations that can be dealt with by Act of Parliament. In bread we find alum, sometimes in crystals, the size of a pea. The alum is used to impart to bread made of flour of second or third rate quality a whiteness which otherwise cannot be obtained except in bread of the first quality, besides which, it enables the baker to force into his bread a larger quantity of water than he could otherwise do; the alum imparts to bread the property of retaining that water after it is taken from the oven. There are sometimes 250 grains of alum in the 4lb. loaf, and this quantity is most injurious to health, and the worst of this adulteration is that it is the poor man, who does not buy the best bread, that suffers. Boiled rice is also used in bread, and introduces into the loaf a large quantity of water. Carbonate of magnesia, chalk, clay, potato pulp, ammonia, beans, bonedust, sulphate of copper, plaster of Paris, soda, zinc (sulphate of), carbonate of magnesia are all little "extras" that may be found with the flour in our breakfast loaf.

Beer is very largely adulterated after it passes out of the hands of the brewer and into that of the publican; there is sometimes a difference of 50 per cent. in the quantity of alcohol which the same beer contains when it comes from the brewery and when it is bought from the publican. Common salt is used for increasing the thirst of the consumer, and sulphate of iron for the purpose of giving froth and "head" to the beer. Cocculus indicus, liquor ammonia, extract of gentian, foots sugar, chloride of sodium, copperas, strychnine, tobacco, opium, alum, picric acid, all go into the publicans' beer barrel. "Cocculus indicus," which is one of the ingredients prohibited by the Act of 1872, is a poisonous substance, a true poison, and contains 3 per cent. of the poison called pitrotoxine, used by poachers for destroying pheasants and game. This stuff is frequently used for the purpose of causing a species of inebriety which should be due to alcohol; it creates a feeling of intoxication without the previous excitement which alcohol produces. "It knocks you down," so to say, without previous exhilaration.

Cheese is seldom adulterated. It is generally only coloured with annatto, saffron, or carrots, but on the continent potatoes and starch are used, and a wash or preparation containing arsenic has been used on the rind of foreign cheeses to preserve them from attacks of fly. In some kinds, sage, parsley, etc., are used in process of manufacture in order to flavour the cheese, and fragments of leaves may therefore be detected. Sweets are coloured green with arsenic (a deadly poison), while in coloured confectionery generally, white potter's clay, and pipe clay, plaster of Paris, and sand are found, and colours like cobalt, smalt, and ultramarine are largely used. The bright reds, greens, and yellows are the most dangerous sweets; all shades of brown up to black are safest, because these are produced by the sugar being partly or wholly changed into caramel. Butter is principally adulterated with water. Really good butter should only contain 10 per cent. of water and 2½ per cent. of salt; but it usually contains 20 per cent. of water, and in fresh Devonshire and Normandy butter there is over 16 per cent. of water. Often 35 and 40 per cent. of water has been found by the analysts. Animal fats are sometimes used, such as lard, beef, mutton, veal, and horse fat. "Bosh" butter is mixed with starch, generally potato flour. French butters are much more adulterated than those made in this country.

Bottled fruits and vegetables are adulterated with certain salts of copper, usually the sulphate or acetate. These colouring matters are highly injurious. Pickles contain substitutions, such as shrivelled cucumbers instead of gherkins, or white cabbage (coloured red) for red cabbage. Anchovies we seldom or ever get; various kinds of fish are substituted for the true or Gorgona anchovy, such as Dutch, French, or Sicilian fish, sardines, and even sprats. It is only by a perfect acquaintance with the characters of the fish that these frauds can be detected. True anchovies come to England in barrels, preserved in strong brine, and then they are bottled by the wholesale pickle merchants. Sprats and other small fish are often sold as sardines.

Sausages bought in large towns in the usual way can never be depended upon. The best portions of horseflesh are sold to mix with collared brawn, and for sausages and polonies. The horseflesh materially assists the making of sausages. It is a hard fibrine, and it mixes better; and it keeps them hard, and so they last longer in a shop window before they are sold, because otherwise the sausages run to water, and become soft and pulpy. Horseflesh is largely used in German sausages. A writer in a popular journal recently affirmed that in London the best sausages were obtained from shops, the proprietors of which did not object to selling to their customers sausage meat, and that sausages obtained from those places where a request for a small quantity of such meat was met with a refusal were invariably bad. Sausages, and more particularly the large German ones, frequently become poisonous from the development of a peculiar rancid, fatty acid, produced during decomposition of the meat. Liebig ascribes the poisonous effects to an animal ferment. The poisonous property is usually observed in April, and for this reason others allege it is due to a fungus, the crytogamic organisms of which are most active in that month. As to the reputed use of our domestic "pussies" and "doggies" for sausage meat it is needless to enquire too closely. Cocoa and chocolate are seldom pure. The former is mixed with sugar, reddle, venetian red, amber chicory, cocoa husk, cereal grains, arrowroot, potato starch, and sago. Chocolate, in addition, with copper, lime, lentils, maize, beans, olive oil, almonds, yolk of egg, veal and mutton fat, benzoin, arabic gum, cinnabar, red earths, red lead, red oxide of mercury.

Tobacco is commonly adulterated with water, gum, saccharine matter and various salts. In cut tobaccos liquorice, gum catechu, salt, saltpetre, and various nitrates, yellow ochre, Epsom salts, Glauber salts, green copperas, red sandstone, wheat, oatmeal, maltcombings, chicory, and the following leaves: coltsfoot, rhubarb, chicory, endive, oak, and elm, and in fancy tobacco lavender and mugwort are found. In roll tobacco rhubarb, endive, and dock leaves, sugar, liquorice, and a dye made from logwood and sulphate of iron have been met with. The cigars sold at fairs and in the streets are seldom anything but brown paper and hay, and are unlikely to deceive anyone but the silly youths that buy them. They are generally steeped in various saccharine and saline infusions. Cheap cigarettes often contain sand.

Gin and whisky are adulterated with alum. Sub-carbonate of potash, acetate of lead, and sulphuric acid, sugar, cayenne, and caustic potassa are added to sweeten, give pungency, and apparent strength, while sliced horseradish and sulphate of zinc are often mixed with the spirit to give it "piquancy" and "mellowness." Cassia buds and orris are added. Brandy is doctored with water, burnt sugar, cayenne pepper, horseradish, grains of paradise, fusel oil, acetic ether. Cheap brandies are made from corn spirit, and flavouring and colouring matter. In the trade the addition of water is known as "reducing," while absolute adulteration is called "improving." Here is a trade receipt: "To ten puncheons of brandy (1,081 gallons) add flavouring raisin spirit (118 gallons), tincture of grains of paradise (four gallons), cherry laurel water (two gallons), spirit of almond cake (two gallons), total 1,207 gallons; add also ten handfuls of oak sawdust and some burnt sugar to give it a complexion."

Preserved fruits, jams, frequently contain copper, and it sometimes is present in large quantities. It is derived either from the copper vessels in which the preserves are often prepared, or it has been added to improve the colour of the article. Sauces, like anchovy essence, lobster, shrimp and tomato sauces, are adulterated with red ferruginous earths as bole, armenian and venetian red. Pepper, vinegar, mustard, are all mixed with foreign substances, while curry powders contain ground rice, potato farina and salt, coloured with red lead. All these various adulterants in our food and our drink have been proved by evidence laid before Parliamentary Committees to exist in the various articles referred to.

